# Impact of Age and Sex on Electrical Impedance Values in Healthy Oral Mucosa

**DOI:** 10.3390/bioengineering9100592

**Published:** 2022-10-21

**Authors:** Kristina Horvat, Ivica Richter, Vesna Vucelić, Krešimir Gršić, Dinko Leović, Ivana Škrinjar, Ana Andabak Rogulj, Marko Velimir Grgić, Vlaho Brailo

**Affiliations:** 1 Department of Oral Medicine, School of Dental Medicine, University of Zagreb, 10000 Zagreb, Croatia; 2 Clinic for Anaesthesiology, Clinical Hospital Centre “Sestre Milosrdnice”, 10000 Zagreb, Croatia; 3 Clinic for Ear, Nose, Throat and Head and Neck Surgery, University Clinical Hospital Centre Zagreb, 10000 Zagreb, Croatia; 4 Clinic for Dentistry, University Clinical Hospital Centre Zagreb, 10000 Zagreb, Croatia; 5 Clinic for Otolaryngologyand Head and Neck Surgery, Clinical Hospital Centre “Sestre Milosrdnice”, 10000 Zagreb, Croatia

**Keywords:** electrical impedance, healthy mucosa, variability, sub pressure, diagnostics

## Abstract

Background: Electrical impedance (EI) is a property of all living tissues and represents the resistance to the electric current flow through a living tissue. EI depends on the structure and chemical composition of the tissue. The aim of this study was to determine the influence of age, sex, and electrode pressure on the EI values of healthy oral mucosa. The study involved 101 participants with healthy oral mucosa who were divided into three age groups. EI was measured in seven anatomical regions. Results: Significant differences between different age groups were found. Younger participants (20–40 years) had significantly higher EI values than the older participants (60+). Significantly higher EI values were found in women at all localisations at all measured frequencies, except on the hard palate. EI values measured with higher sub-pressure were significantly lower than values measured with lower sub-pressure at all frequencies and localisations, except the tongue dorsum, tongue border, and sublingual mucosa. Conclusions: This study found that EI values in healthy oral mucosa depend on age and sex and may also depend on the pressure of the measuring device. These factors should be kept in mind when EI is used as a diagnostic method for different oral lesions.

## 1. Introduction

Electrical impedance (EI) is the resistance to the electric current flow through a living tissue. Every living tissue has its own EI spectrum which is determined by its structure and chemical composition. Structural and/or chemical changes in tissue such as inflammation, ischemia, necrosis, and disruption of the basal membrane result in altered EI values. The application of EI for diagnostic purposes in medicine and dentistry is based on this property [1,2,3]. EI-based methods have a certain advantage over invasive methods such as less discomfort for the patient and less complicated disinfection, sterilisation, and infection control procedures [4,5,6].

The most widely used EI-based diagnostic method is EI spectroscopy. The method was first used in dermatology for the assessment of different skin lesions such as atopic dermatitis, contact allergy, and skin irritations [7,8,9,10]. Nyren and co-workers [8] reported on the significantly decreased values of EI in irritative dermatitis compared to contact dermatitis. Nicander and co-workers [9] reported significantly different EI values between healthy skin and clinically unchanged skin in patients with atopic dermatitis. Apart from different inflammatory skin conditions, EI is mostly used as an adjunct in diagnostics of different benign and malignant skin tumours [10,11,12]. Apart from its use in dermatology, EI has been investigated as a diagnostic technique for the detection of breast cancer as well as a method for the assessment of lean muscle loss and cachexia [13,14]. 

In dentistry, the most widespread application of EI is in root canal length measuring devices [15,16], and it is used for diagnostic purposes in oral mucosa under investigation. In their pioneering study, Nicander et al. [17] measured EI on oral mucosa and reported significant differences between healthy mucosa and chemical-agent-induced mucosal irritations. Other investigators found significantly different EI spectra in oral cancer, oral potentially malignant disorders, and healthy oral mucosa [18,19,20]. 

The question that arises is whether there any individual factors that might affect healthy mucosa EI values in the absence of an obvious clinical pathology. In their pilot study, Richter et. al. [21] measured EI spectra of healthy oral mucosa and the factors that affect it. EI values varied between different anatomical regions, and it was reported that the factor mostly influencing EI values was the degree of mucosal keratinisation. Thus, the highest EI values were measured on the hard palate, and the lowest on the tongue dorsum. EI values on the hard palate were significantly higher than the values in other regions, whereas EI values between other regions were not significantly different. The EI values measured on the left and right side in the same region did not differ significantly. Smokers had somewhat higher EI values than non-smokers, but the differences were significant only on upper lip mucosa at frequencies of 50, 70, and 100 kHz. No correlation was found between EI and salivary flow at any of the sites. 

In that study, the authors acknowledged several factors that might affect EI values, such as age, sex, and the pressure of the intraoral sensor. It is well known that oral mucosa undergoes atrophy with ageing, and these ultrastructural changes might affect the resistance to the electric current flow [22,23,24,25]. The age span of participants in a study by Richter et al. was rather narrow (20–40 years), and no conclusions regarding the impact of age on EI in healthy oral mucosa could be drawn [21]. Even though a difference in EI between males and females was observed in several regions (upper lip, tongue dorsum), no conclusion could be drawn due to a small number of participants. The authors also identified the stability of the intraoral sensor as one of the factors that might have an impact on EI values. Therefore, the aim of this study was to compare EI values between males and females of different age groups. Furthermore, the aim was also to assess if the pressure of a measuring device might have an impact on EI measurements in healthy oral mucosa. 

## 2. Materials and Methods

The study was approved by the Ethics Committee of the School of Dental Medicine University of Zagreb (approval no. 05-PA-26-22/12). Before the recruitment, the details of participation in the study were explained to all participants in oral and written form. All inquiries by participants were answered by the researchers. Before joining the study, the participants signed the Informed Consent Form, which was produced in accordance with the Declaration of Helsinki. The study involved 101 participants with healthy oral mucosa who were divided into three age groups: the first group consisted of 42 participants aged 20–39 years, the second group consisted of 30 participants between the ages of 40 and 59, and the third group consisted of 29 participants between the ages of 60 and 80. Criteria for the selection of the participants were as follows: clinically healthy oral mucosa and understanding of the informed consent form. 

**Figure 1:**
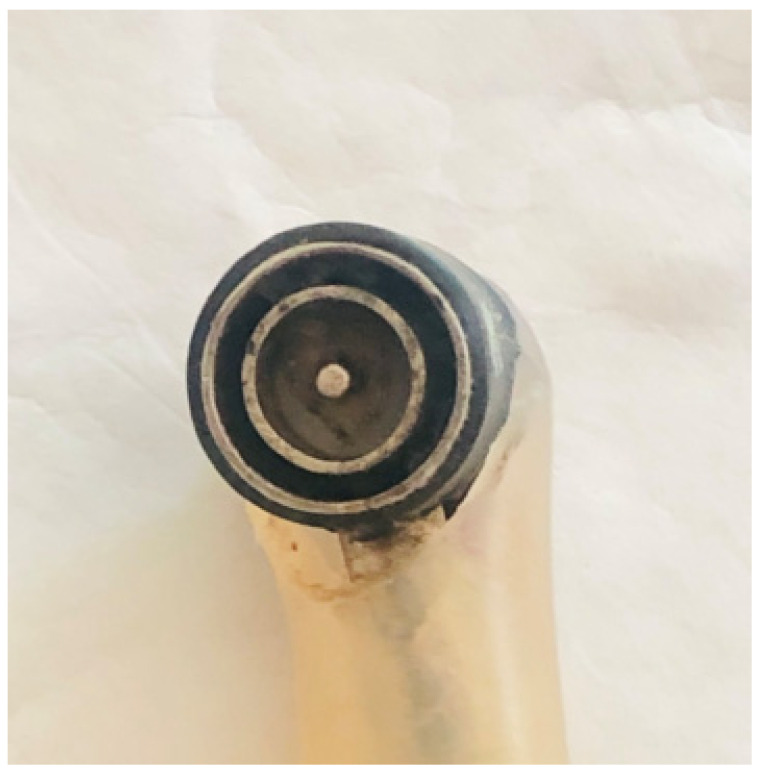
Intraoral probe consisting of 3 concentric aluminium alloy electrodes.

Device for the measurement of EI consisted of three parts: intraoral probe, measurement instrument (NI USB-6251 (National Instruments^®^, Austin, TX, USA), and a laptop [19]. The intraoral probe was fabricated from three concentric high-conductivity sintered aluminium alloy electrodes (total diameter of 8 mm), which were coated with the isolation layer of Teflon (Figure 1). To assure the probe stability and constant pressure on oral mucosa, the probe was connected to a dental suction which produced sub-pressures of 250 mBar and 350 mBar, respectively. The probe was connected to the measuring device by electric conductors, and the device was attached through USB connection with a laptop. Electric conductors were used to connect the intraoral probe with the measuring device, while an USB connection was used to connect the measuring device and a laptop. A Lab-View-based software (Lab View 8.5.1. National Instruments^®^, Austin SAD) was used to convert measurements into digital records, which were stored in an Excel^®^ worksheet.

EI was measured in 14 points (bilaterally in 7 anatomical regions of the oral cavity, i.e., in the upper lip mucosa, lower lip mucosa, hard palate, buccal mucosa, tongue dorsum, ventral tongue, and sublingual mucosa). The measurement was performed by placing the probe against the selected point on the mucosa. When the sub pressure of 250 mB was reached, which was confirmed by the vacuum gauge (Yuyao Yadong Plastic, Huangzhou, China), the device was turned on, and the EI for the respective point was registered through nine frequencies (1, 2, 5, 7, 10, 20, 70, and 100 kHz). The measurement was first performed with a probe sub pressure of 250 mBar, and after that, the whole procedure was performed with a sub pressure of 350 mBar. All measurements were performed by a single examiner. Three sets of measurements were performed for each participant, and the average EI value was used for further calculations.

SPSS software (IBM Inc., Armonk, NY, USA) was used for statistical analysis. The Kolmogorov–Smirnov test was used to assess normality of distribution. Difference between categorical variables was assessed by chi-squared test. Student’s *t*-test and analysis of variance (ANOVA) with post hoc Bonferroni test (where appropriate) were used to examine differences between linear variables. The intra-class correlation coefficient was used to assess intra-rater agreement. *p*-values lower than 0.05 were considered statistically significant. 

## 3. Results

A total of 101 subjects (56 women and 45 men) with an average age of 46.15 ± 19.53 years participated in the study. Among the participants, there were 78 (77.2%) non-smokers and 23 smokers (22.8%). Participants’ demographic data are presented in Table 1. 

No significant difference in the proportion of men and women between the groups was found. No significant difference in the prevalence of smokers and non-smokers was found between the groups. No significant difference in salivary flow was found between the groups.

**Figure 2:**
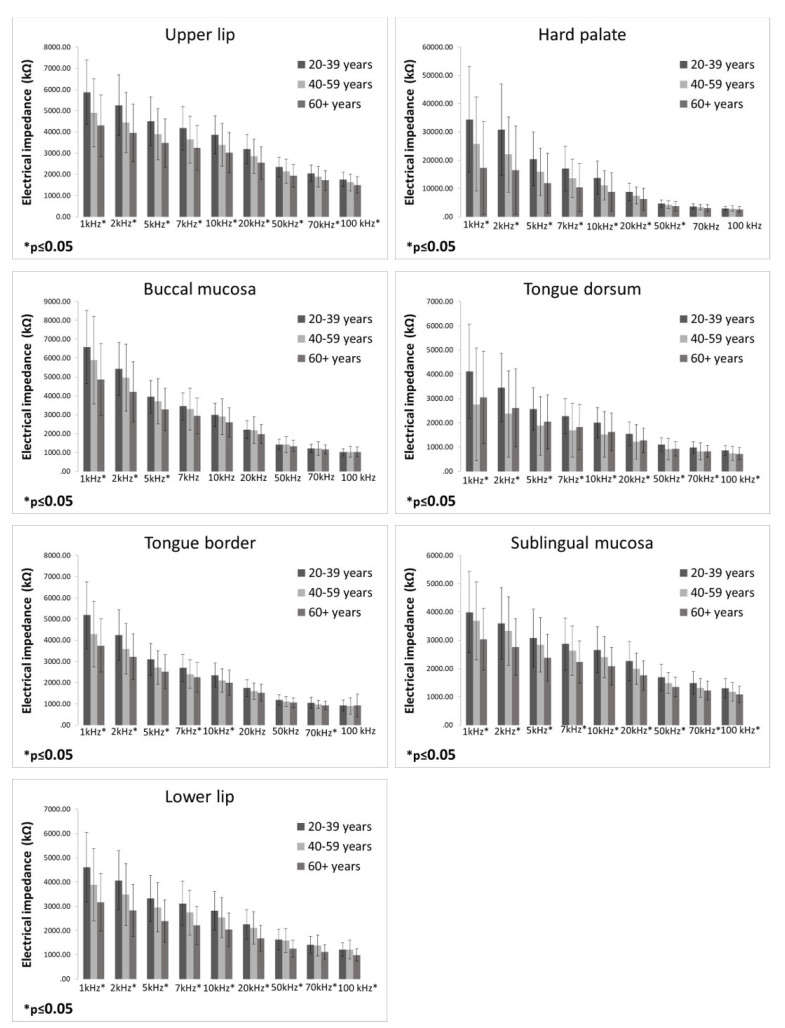
Electrical impedance measurements in three different age groups (bars represent mean ± standard deviation).

EI measurements in three different age groups are presented in Figure 2. The highest EI values were found in participants aged 20–39 years compared to participants aged 40–59 years and 60 years and over, respectively. Participants aged 20–39 years had significantly higher EI values than participants aged 60 and more years at all measured frequencies on the upper lip, tongue dorsum, sublingual mucosa, and lower lip. On the hard palate, a significant difference between these two groups was found at all measured frequencies except 70 and 100 kHz. On the buccal mucosa, participants aged 20–39 years had significantly higher EI values compared to participants aged 60 years and over at frequencies of 1, 2, and 5 kHz. On the tongue border, participants aged 20–39 years had significantly higher EI values compared to participants aged 60 years and over at all frequencies except 20, 50, and 100 kHz. Furthermore, participants aged 20–39 years had significantly higher EI values compared to participants aged 40–59 on the upper lip at the frequency of 1 kHz; on the tongue dorsum at all measured frequencies; on the tongue border at the frequency of 1 kHz; and on the lower lip at the frequencies of 10, 20, 50, 70, and 100 kHz.

**Figure 3:**
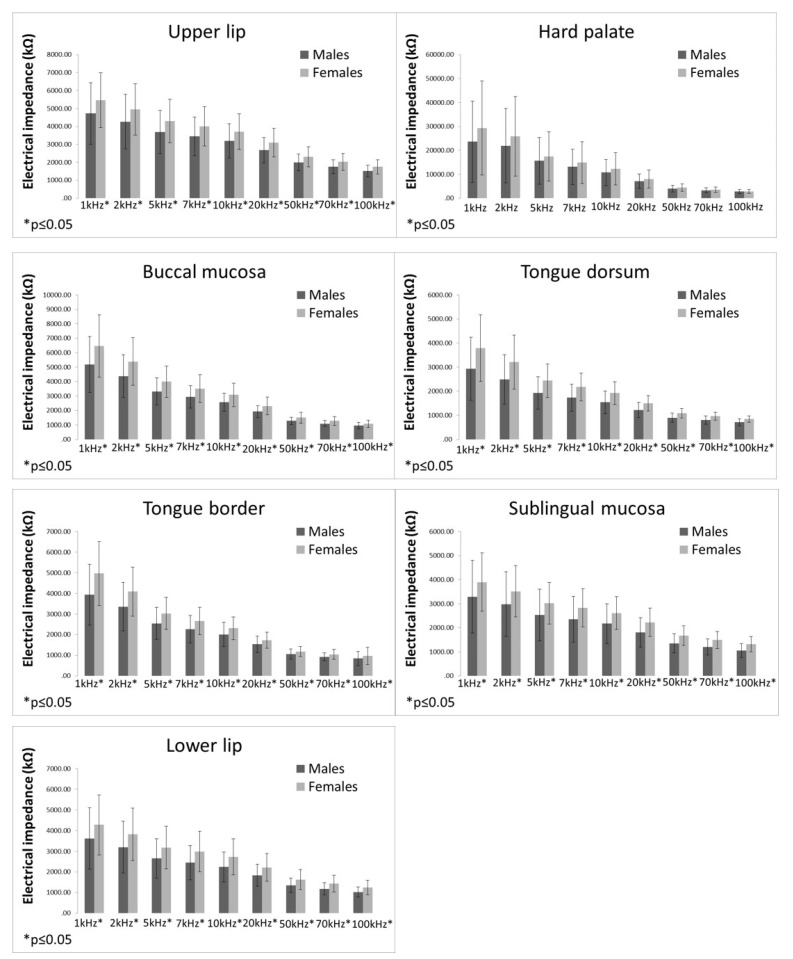
Electrical impedance measurements in males and females (bars representing mean ± standard deviation).

The difference in EI values between males and females are presented in Figure 3. Females had significantly higher EI values in all measured localisations at all measured frequencies than men, except for the hard palate. In the hard palate, no significant differences between males and females were detected for any of the measured frequencies. 

**Figure 4:**
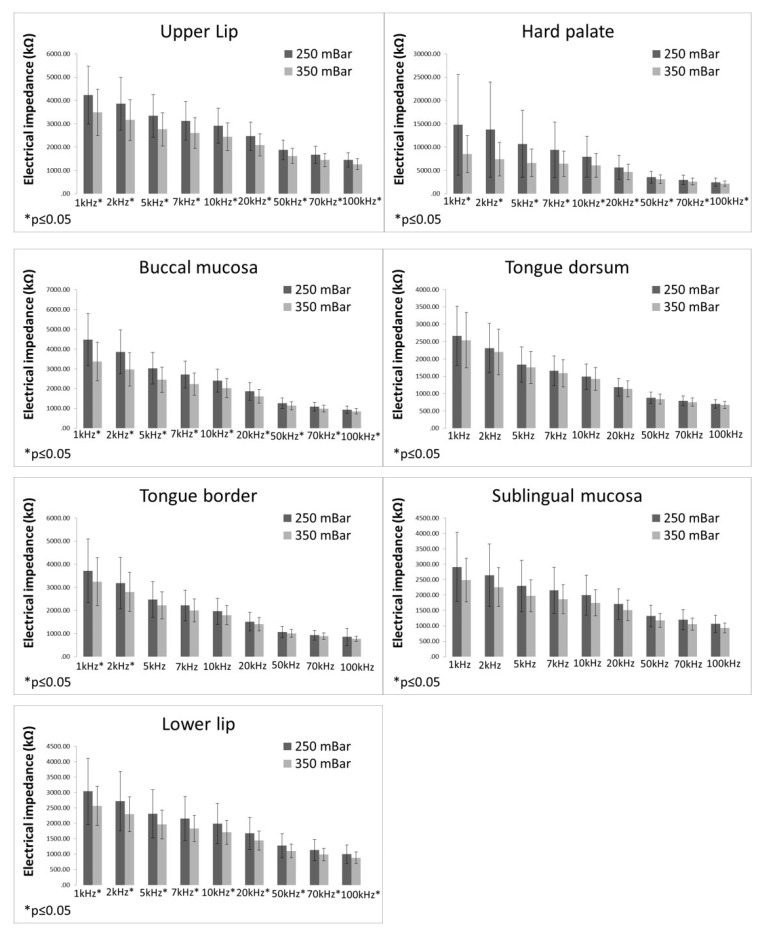
Electrical impedance measurements with different sub-pressures (bars representing mean ± standard deviation).

EI values measured with different sensor sub-pressure (250 vs. 350 mBar) are displayed in Figure 4. EI values measured at the sub-pressure of 250 mBar were significantly higher at all frequencies than EI values measured at the sub-pressure of 350 mBar at the upper and lower lips, hard palate, and buccal mucosa. On the tongue dorsum, no significant differences between EI values measured at 250 mBar and 350 mBar were found. On the tongue border, EI values measured at 250 mBar were significantly higher than EI values measured at 350 mBar only at the frequencies of 1 and 2 kHz. In the sublingual mucosa, EI values measured at 250 mBar were significantly higher than EI values measured at 350 mBar only at the frequency of 100 kHz.

Intra-rater agreement assessed by the intra-class correlation coefficient was found to be good to very good (0.58–0.81) depending on the frequency and the location of the measurement point indicating good reproducibility of the measurement(s). Intra-class correlation coefficient values are displayed in Table 2 (for the sub pressure of 250 mBar) and Table 3 (for the sub pressure of 350 mBar).

## 4. Discussion

The aim of this study was to determine the influence of age, sex, and electrode pressure on the EI values of healthy oral mucosa. Significant differences between different age groups were found. Younger participants (20–40 years) had significantly higher EI values than the older participants (60+) at all localisations at almost all measured frequencies. Compared to middle-aged participants (40–60 years), younger participants also had higher EI values, but a significant difference was found only at certain frequencies and most localisations (upper lip, cheek, dorsum, tongue edge, lower lip). This finding can be interpreted primarily by involutional changes in older subjects. Hormonal and atrophic changes in the mucosa are known to occur with age [22,23,24,25,26]. The structure of the mucosa weakens with the weakening of intercellular connections, which leads to an increase in extracellular space. With the increase in extracellular space, there is a decrease in tissue resistance due to easier penetration of current into the extracellular fluid occurs [13,27,28]. There are no similar studies on oral mucosa for comparison. However, our results are in concordance with the results of Nicander et al. [17] in a work conducted on the skin where the age was shown to significantly affect the decrease in EI magnitude in the elderly subjects.

In this study, significantly higher EI values were found in women at all localisations at all measured frequencies except on the hard palate. However, it should be noted that on the hard palate, EI values in women were higher than in men, despite the absence of significant differences. It is possible that these differences were due to the protective action of female sex hormones. Thanks to higher production of oestrogen (17 beta oestradiol), women have more robust humoral and cellular immunity than men, which promotes faster regeneration of immune, epithelial, and muscle cells that make the mucosa more resistant to various noxes, as well as possibly to the flow of electric current [29,30,31,32]. Regarding the impact of sex on EI in the oral mucosa, there are no data for comparison with our results. On the other hand, results of EI measurements on the skin are conflicting. Thus, Nicander et al. [9], in a study of 131 participants, argue that gender differences are only of marginal importance, while Aberg [10], using different statistical methods on the full EI spectrum, concludes that gender has a “dramatic impact” and should be taken into account when planning studies of EI. 

In this study, a vacuum was used to stabilise the measuring electrode. The intention to introduce a vacuum-fixed electrode was to eliminate the influence of the examiner (hand tremor, etc.) in order to standardise the adhesion and contact of the electrode with the mucosa. In order to optimise the measurement procedure, measurements were performed with two different sub-pressures (250 and 350 mBar) in order to determine how the difference in the electrode pressure can affect the results. EI values measured with higher sub-pressure were significantly lower than values measured with lower sub-pressure at all frequencies and localisations, except the tongue dorsum, tongue border, and sublingual mucosa. This finding can be primarily explained by the fact that the measuring electrodes were in closer contact with the tissue at these localisations. The mucosa in these sites provides less resistance to the sub-pressure, which can affect the adhesion of the electrode and lead to a more uniform measurement. Uniform pressure and complete stabilisation of the electrode is not completely possible if the electrodes are placed only by the force of the examiner’s hand, and as can be seen from our results, they can significantly affect the EI values. Our results cannot be compared to other studies, since to our knowledge, no other systems have used sub-pressure for the fixation of the measuring electrode. The method displayed good reproducibility as can be seen form the intra-examiner statistics. 

The application of EI for diagnostics of different oral mucosal pathologies is to our knowledge focused on oral cancer and oral potentially malignant disorders (OPMD), with these conditions having been intensively investigated. Tatullo et al. [18] determined significantly different EI values between healthy individuals and patients with oral lichen planus. In their preliminary study in oral cancer patients, Ching et al. [4] measured significantly lower EI values compared to healthy, clinically unaffected mucosa surrounding the tumour. Sarode et al. [19] compared EI in 50 cancer patients and 50 healthy controls, reporting that oral cancer patients had a significantly lower EI value than healthy individuals. Using a commercially available EI-based device (developed for the diagnosis of cervical cancer), Murdoch et al. [20] found significantly different EI spectra in oral cancer, oral potentially malignant disorders, and healthy oral mucosa. What can be concluded from the aforementioned studies is that healthy oral mucosa has its own EI spectrum which is different from the spectra of different mucosal pathologies. On the basis of the results from this study, we found that spectrum is dependent on individual factors such as age and sex, which need to be taken into account when assessing the diagnostic potential of EI. 

The main limitation of this study is the fact that the total number of participants was relatively small, and it is therefore questionable as to how much the results can be generalised to the whole population. 

## 5. Conclusions

This study found that EI values in healthy oral mucosa depend on age and sex, and may also depend on the pressure of the measuring device. In order to use EI as a diagnostic method for oral lesions, a range of EI values for healthy oral mucosa in different age groups needs to be determined (reference values). These studies need to include a higher number of individuals of different ages and sex in whom EI needs to be measured on several occasions in order to register intra- and inter-individual differences. Furthermore, an intraoral sensor needs to be constructed in a way that assures constant pressure of the measuring electrode on the oral mucosa. Along with that, optimal sub-pressure that assures the highest intra-rater agreement should be identified.

## Figures and Tables

**Table 1:** Demographic data on participants.

	Total	Age 20–39	Age 40–59	Age > 60	p
Sex N (%)					
Male	45 (44.6)	17 (40.5)	16 (55.2)	12 (41.4)	0.271
Female	56 (55.4)	25 (59.5)	14 (44.8)	17(58.6)	
					
Age (average ± SD)	46.15 ± 19.53	26.64 ± 4.72	48.9 ± 6.18	71.48 ± 6.75	/
					
Smoking N (%)					
Yes	23 (22.8)	12 (28.6)	8 (24.1)	4 (13.8)	0.343
No	78 (77.2)	30 (71.4)	22 (75.9)	25 (86.2)	
					
Salivary flow mL/min (average ± SD)	0.46 ± 0.18	0.52 ± 0.22	0.53 ± 0.21	0.4 ± 0.24	0.052

**Table 2:** Intra-rater statistics for measurements at the sub pressure of 250 mBar.

Upper Lip									
Frequency	1 kHz	2 kHz	5 kHz	7 kHz	10 kHz	20 kHz	50 kHz	70 kHz	100 kHz
ICC	0.752	0.733	0.685	0.693	0.699	0.705	0.703	0.701	0.698
p	0.000	0.000	0.000	0.000	0.000	0.000	0.000	0.000	0.000
Hard Palate									
Frequency	1 kHz	2 kHz	5 kHz	7 kHz	10 kHz	20 kHz	50 kHz	70 kHz	100 kHz
ICC	0.555	0.541	0.654	0.666	0.688	0.713	0.677	0.659	0.580
p	0.000	0.000	0.000	0.000	0.000	0.000	0.000	0.000	0.000
Buccal Mucosa									
Frequency	1 kHz	2 kHz	5 kHz	7 kHz	10 kHz	20 kHz	50 kHz	70 kHz	100 kHz
ICC	0.722	0.694	0.674	0.681	0.678	0.628	0.643	0.647	0.443
p	0.000	0.000	0.000	0.000	0.000	0.000	0.000	0.000	0.000
Buccal Mucosa									
Frequency	1 kHz	2 kHz	5 kHz	7 kHz	10 kHz	20 kHz	50 kHz	70 kHz	100 kHz
ICC	0.763	0.813	0.801	0.798	0.793	0.787	0.779	0.775	0.777
p	0.000	0.000	0.000	0.000	0.000	0.000	0.000	0.000	0.000
Tongue Border									
Frequency	1 kHz	2 kHz	5 kHz	7 kHz	10 kHz	20 kHz	50 kHz	70 kHz	100 kHz
ICC	0.681	0.659	0.627	0.634	0.637	0.651	0.666	0.463	0.118
p	0.000	0.000	0.000	0.000	0.000	0.000	0.000	0.000	0.221
Sublingual Mucosa									
Frequency	1 kHz	2 kHz	5 kHz	7 kHz	10 kHz	20 kHz	50 kHz	70 kHz	100 kHz
ICC	0.652	0.629	0.657	0.665	0.676	0.676	0.697	0.679	0.596
p	0.000	0.000	0.000	0.000	0.000	0.000	0.000	0.000	0.000
Lower Lip									
Frequency	1 kHz	2 kHz	5 kHz	7 kHz	10 kHz	20 kHz	50 kHz	70 kHz	100 kHz
ICC	0.749	0.703	0.713	0.675	0.687	0.692	0.679	0.673	0.655
p	0.000	0.000	0.000	0.000	0.000	0.000	0.000	0.000	0.000

**Table 3:** Intra-rater statistics for measurements at the sub pressure of 350 mBar.

Upper Lip									
Frequency	1 kHz	2 kHz	5 kHz	7 kHz	10 kHz	20 kHz	50 kHz	70 kHz	100 kHz
ICC	0.729	0.708	0.691	0.686	0.682	0.674	0.667	0.668	0.676
p	0.000	0.000	0.000	0.000	0.000	0.000	0.000	0.000	0.000
Hard Palate									
Frequency	1 kHz	2 kHz	5 kHz	7 kHz	10 kHz	20 kHz	50 kHz	70 kHz	100 kHz
ICC	0.646	0.698	0.691	0.644	0.615	0.691	0.712	0.808	0.798
p	0.000	0.000	0.000	0.000	0.000	0.000	0.000	0.000	0.000
Buccal Mucosa									
Frequency	1 kHz	2 kHz	5 kHz	7 kHz	10 kHz	20 kHz	50 kHz	70 kHz	100 kHz
ICC	0.683	0.684	0.697	0.702	0.703	0.698	0.680	0.673	0.669
p	0.000	0.000	0.000	0.000	0.000	0.000	0.000	0.000	0.000
Tongue Dorsum									
Frequency	1 kHz	2 kHz	5 kHz	7 kHz	10 kHz	20 kHz	50 kHz	70 kHz	100 kHz
ICC	0.710	0.734	0.749	0.755	0.756	0.753	0.737	0.730	0.720
p	0.000	0.000	0.000	0.000	0.000	0.000	0.000	0.000	0.000
Tongue Border									
Frequency	1 kHz	2 kHz	5 kHz	7 kHz	10 kHz	20 kHz	50 kHz	70 kHz	100 kHz
ICC	0.741	0.733	0.693	0.674	0.653	0.611	0.562	0.543	0.540
p	0.000	0.000	0.000	0.000	0.000	0.001	0.002	0.003	0.003
Sublingual Mucosa									
Frequency	1 kHz	2 kHz	5 kHz	7 kHz	10 kHz	20 kHz	50 kHz	70 kHz	100 kHz
ICC	0.506	0.505	0.525	0.541	0.552	0.572	0.582	0.579	0.570
p	0.006	0.006	0.004	0.003	0.002	0.001	0.001	0.001	0.002
Lower Lip									
Frequency	1 kHz	2 kHz	5 kHz	7 kHz	10 kHz	20 kHz	50 kHz	70 kHz	100 kHz
ICC	0.732	0.722	0.704	0.697	0.687	0.660	0.606	0.582	0.446
p	0.000	0.000	0.000	0.000	0.000	0.000	0.001	0.001	0.019

## Data Availability

The data presented in this study are available on request from the corresponding author.
